# Chromosome Genome Assembly of the Leopard Coral Grouper (*Plectropomus leopardus*) With Nanopore and Hi-C Sequencing Data

**DOI:** 10.3389/fgene.2020.00876

**Published:** 2020-09-02

**Authors:** Yongbo Wang, Xin Wen, Xinhui Zhang, Shuyuan Fu, Jinye Liu, Wei Tan, Ming Luo, Longlong Liu, Hai Huang, Xinxin You, Jian Luo, Fuxiao Chen

**Affiliations:** ^1^Key Laboratory of Utilization and Conservation for Tropical Marine Bioresources of Education of Ministry, Hainan Academy of Ocean and Fisheries Sciences, Hainan Tropical Ocean University, Haikou, China; ^2^State Key Laboratory of Marine Resource Utilization in South China Sea, Hainan Aquaculture Breeding Engineering Research Center, Hainan University, Haikou, China; ^3^Shenzhen Key Lab of Marine Genomics, Guangdong Provincial Key Lab of Molecular Breeding in Marine Economic Animals, BGI Academy of Marine Sciences, BGI Marine, Shenzhen, China

**Keywords:** chromosome-level genome, *Plectropomus leopardus*, leopard coral grouper, Nanopore sequencing, Hi-C technologies

## Introduction

As the storehouse of life information, the genome of an organism harbours all of its biological aspects and evolutionary history. Research conducted at the genomic level has become more common, providing important breakthroughs for the comprehensive interpretation of species. In this respect, numerous, large populations of fish species live in the diverse habitats worldwide, and their genome information presents a valuable genetic resource for fisheries. Exploring the massive genetic information contained in the genomes of fishes can not only reveal the adaptive mechanisms of these organisms to various aquatic habitats, but also help to clarify the gene regulatory networks and mechanisms of the economically relevant traits and important life history phenomena.

Over the past decade, researchers have revealed much fish genome information and associated characteristics (You et al., 2020). For example, whole genome sequencing of Atlantic cod (*Gadus morhua*) revealed its special immune mechanism (Star et al., 2011), and likewise demonstrated the doubling mechanism of the Atlantic salmon (*Salmo salar*) genome (Lien et al., 2016). Analysis of the *Paralichthys olivaceus* genome shows that retinoic acid plays an important role in its eye movement and metamorphic development, achieved via the double antagonistic regulation of thyroxine and retinoic acid (Shao et al., 2017). In constructing the whole genome fine map of channel catfish (*Ietalurus punetaus*), Liu et al. (2016) uncovered the mechanism of its scale formation, and a study of the whole genome of *Leuciscus waleckii* elucidated its alkaline environment adaptation mechanism (Xu et al., 2017), to name a few impressive cases. Besides providing insight to molecular mechanisms underpinning biological characteristics, decoding genome information of fish could be used to lay a sound theoretical foundation for distinguishing the genomic location of key economic traits. For example, based on genome-wide association analysis, disease resistance characters of channel catfish were mapped (Geng et al., 2015), and the SNP (single nucleotide polymorphism) loci related to fat content characters of carp were found by GWAS (Genome-Wide Association Studies) analysis (Zheng et al., 2016). In terms of their breeding, genomic selection and breeding technologies on growth and disease resistance, respectively, have been carried out for economic fish species such as Atlantic salmon (*Salmo salar*) (Ødegård et al., 2014), rainbow trout (*Oncorhynchus mykiss*) (Vallejo et al., 2016) and European sea bass (*Dicen trarchus labrax*) (Palaiokostas et al., 2018). In sum, harnessing genomic information can provide an efficient platform for the in-depth study of the biological and economic characteristics of fish.

The leopard coral grouper, *Plectropomus leopardus*, belongs to the Serranidae family of Perciformes (Morris et al., 2000). It is an important commercial marine fish, being both delicious as sea food and colourful as an aquarium fish (Greenfiel, 2002). This species, due to the high economic price it commands, has been overfished and is now considered under threat by the International Union for Conservation of Nature (IUCN) (Morris et al., 2000). Currently, the genetic resources of the fish are still scarcely known, which greatly hinders both the study and conservation of this species (Wang et al., 2015). Like other coral reef fishes, the leopard coral grouper is capable of displaying a variety of body colours (Wu et al., 2016), which can change rapidly in response to light, food, disease and other stresses (Kingsford, 1992; Wang et al., 2015). In our view, it is a perfect representative model for studying the genetic mechanism of body colouring in coral reef fishes. Moreover, the leopard coral grouper can be used as a material for better understanding the mechanism of melanoma (Lerebours et al., 2016), and for gauging the impact of global warming on coral reef ecosystems (Messmer et al., 2017). The decoding of *P. leopardus*'s genome information could yield insight into its ecological significance and accelerate its genetic breeding applications.

In this study, we provide the chromosome-level genome assembly of leopard coral grouper by using Nanopore sequencing and high-throughput chromosome conformation capture (Hi-C) technologies. Our intent is to illustrate and decipher the genome information of a leopard coral grouper and lay a theoretical foundation for the analysis of its body-colour mechanism. This genome resource will be useful for the future conservation, molecular breeding, and population genetics of the leopard coral grouper.

## Materials and Methods

### Sample Collection, Library Construction, and Sequencing

We collected a female leopard coral grouper from the Qionghai Breeding Base of the Hainan Academy of Ocean and Fisheries Sciences, in Qionghai, China.

To extract DNA from its muscle tissue and blood, a DNA Extraction Kit was used following the manufacturer's protocols. Both the quantity and quality of DNA were determined using a NanoDrop 2000 spectrophotometer (Thermo Fisher Scientific, Wilmington, DE, USA).

Two paired-end libraries (insert sizes of 500 and 800 bp) were constructed according to standard Illumina procedures. These libraries were sequenced using the HiSeq 2,500 platform (Illumina, San Diego, CA, USA) with the PE 150 bp model. The raw data had any adapters and low-quality reads removed by SOAPfilter (Luo et al., 2012). All the ensuing clean reads were then applied to estimate the genome size of the leopard coral grouper through a k-mer analysis, done in the Genome Characteristics Estimation (GCE) software (Liu et al., 2013).

For each Nanopore library, the gDNA was size-selected (10–50 kb) with a Blue Pippin system (Sage Science, USA) and processed using the Ligation Sequencing 1D kit (SQKLSK109, Oxford Nanopore Technologies, UK) according to the manufacturer's instructions. Library construction and sequencing were done by the GridION X5/PromethION sequencer (Oxford Nanopore Technologies, UK) at the Genome Center of Nextomics (Wuhan, China). Base calling was performed on fast 5 files by using the ONT Albacore software (v1.2.6) (Sutton et al., 2019), and only those “passed filter” reads representing data of generally higher quality were used for further analyses.

The Hi-C technique has been used to construct a chromosome-level scaffold (Dudchenko et al., 2017). Our Hi-C library was constructed according to previously reported procedures (Rao et al., 2014). First, we used formaldehyde to fix the conformation of the HMW gDNA. Then, the fixed DNA was sheared with the MboI restriction enzyme; the 5' overhangs induced in that shearing step were then repaired using biotinylated residues. Following the ligation of blunt-end fragments *in situ*, the isolated DNA was reverse-crosslinked, purified and filtered to remove biotin-containing fragments. Next, DNA fragment end repair, adaptor ligation and polymerase chain reaction (PCR) were performed successively. Finally, the Hi-C raw data were sequenced on the Illumina HiSeq X platform in its 150 bp PE mode.

For the gene annotation of leopard coral grouper genome, transcriptome sequencing was carried out with the muscle tissue of *P. leopardus*. The total RNA was extracted using a Trizol reagent (Invitrogen, Carlsbad, CA, USA) and purified using an RNeasy Animal Mini Kit (Qiagen, Valencia, CA) according to the manufacturer's instructions. Agilent 2,100 (Agilent Technologies, Palo Alto, CA) was applied to determine the RNA concentration and the RNA integrity number (RIN). The cDNA library was constructed following the manufacturer's instructions (Illumina, San Diego, CA). Finally, the library was sequenced on a HiSeq 2,500 platform (Illumina, San Diego, CA) using paired-end 150 bp reads. The clean data were obtained by removing reads containing adapters and low-quality reads (e.g., N more than 5% and the quality value <20) from the raw data.

### Genome Assembly and Chromosome Anchoring

**Figure 1:**
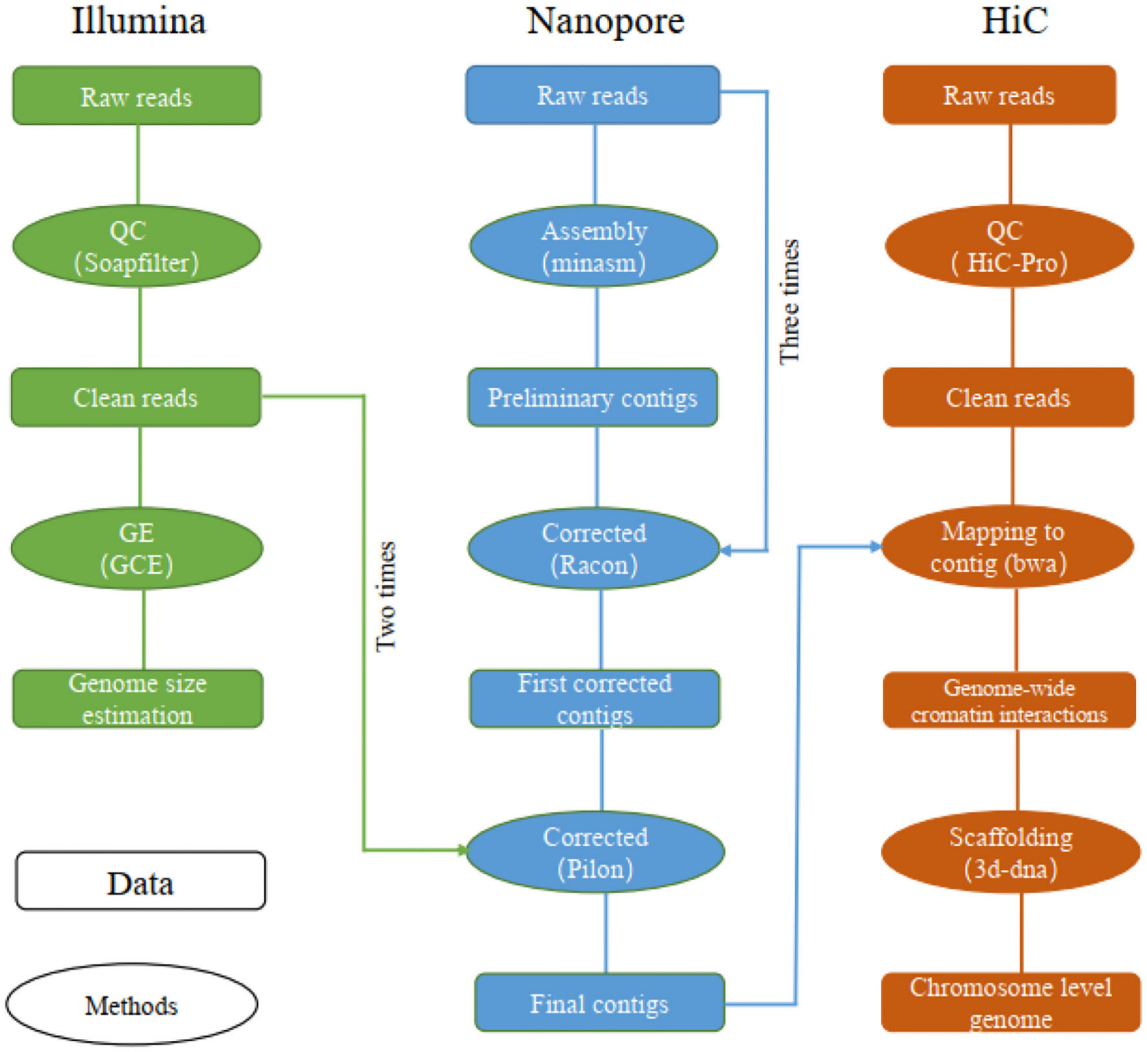
The pipelines used for chromosome-level genome assembly of the leopard coral grouper fish.

Reads obtained from the Illumina reads, Nanopore sequencing data, and Hi-C reads of libraries were used separately for different assembly stages (Figure 1). Specifically, the Illumina reads, Nanopore sequencing data and Hi-C reads were obtained for genome size estimation, *de novo* contig assembly, primary scaffolding, genome survey and sequence error-correction, contig assembly and chromosome anchoring, respectively.

The long reads were assembled using “minimap2” (v2.14) and “miniasm” (v0.3) under their default parameters (Li, 2016, 2018). The assembled contigs were corrected by “racon” v1.3.1 by using long reads (repeated three times) (Vaser et al., 2017), followed by two rounds of polishing by “pilon” v1.22 using the Illumina reads (Walker et al., 2014; Michael et al., 2018).

To obtain the chromosome-level genome, we constructed an interaction matrix with the cleaned reads from the Hi-C library by using HiC-Pro (v2.8.0, default parameters and LIGATION_SITE=GATC) (Servant et al., 2015); this was mapped to the *de novo* assembled contigs to construct contacts among the contigs in “bwa” (v0.7.15) with its default parameters (Li and Durbin, 2009). The bam files containing Hi-C linking messages were processed by another round of filtering, in which any reads were removed if they did not map to the assembled genome within 500 bp from the nearest restriction enzyme site (“juicer” v1.7) (Durand et al., 2016). To assemble the chromosome-level genome based on genomic proximity signals in the Hi-C data, the 3d-dna (v170123) pipeline was used with parameters set to ′-m haploid -s 0 -c 24′ (Sutton et al., 2019).

### Genomic Quality Assessment

To evaluate the quality of the assembled genome, its completeness and accuracy were assessed by using short-read mapping and BUSCO (v3.1) (Simão et al., 2015). We aligned Illumina short reads to the genome by using “bwa” (v0.7.15) (Li and Durbin, 2009).

### *De novo* Repeat Sequences and Gene Annotation

The repeat sequences in the leopard coral grouper genome were identified using a combination of homology-based and *de novo* approaches. First, the homology-based approach was detected repeat sequences, after which the Tandem Repeats Finder (version 4.07) was applied to search for tandem repeats (Benson, 1999). Then, RepeatMasker (v4.0.6) and RepeatProteinMask (v4.0.6), with updated software from the RepeatMasker package), were used jointly to detect known transposable elements (TEs) based on the Repbase TE library (v 21.01) (Tarailo-Graovac and Chen, 2009; Bao et al., 2015). Next, RepeatModeler (v1.0.8) and LTR_FINDER (v1.0.6) set to their default parameters were used to generate the *de novo* repeat library (Xu and Wang, 2007), after which RepeatMasker (Tarailo-Graovac and Chen, 2009) was relied upon again to search for repeat regions against the built repeat library.

### Gene Structure Prediction

For gene structure annotations, we utilised three different approaches to annotate the structures of predicted genes in our assembly genome, including *de novo* prediction, homology-based prediction, and transcriptome-based prediction. For *de novo* predictions, both AUGUSTUS (v3.2.1) (Burge and Karlin, 1997; Stanke et al., 2006) and SNAP (v1.0) (Korf, 2004) software packages were used to identify pro-coding genes within the leopard coral grouper genome. For homology-based predictions, we aligned the homologous proteins of eight fish species—*Danio rerio, Gasterosteus aculeatus, Oreochromis niloticus, Oryzias latipes, Takifugu rubripes, Lepisosteus oculatus, Epinephelus tauvina* and *Lates calcarifer* (from the “ensembl 97” release) (Hubbard et al., 2005)—to the repeat-masked genome by using the “tblastn” tool (Blastall v2.2.26) (Mount, 2007) at threshold cut-off E-value ≤ 1e-5. Next, the Solar (v0.9.6) (Li et al., 2010) and GeneWise (version 2.4.1) (Birney et al., 2004) programs were executed to distinguish and delineate the potential gene structures for all alignments made. Additionally, the RNA-Seq data from muscle tissues were aligned to the assembled genome by using “tophat” (v2.0.13) (Trapnell et al., 2009), and their corresponding gene structures were predicted by “cufflinks” (v2.1.1) (Trapnell et al., 2012). The above three datasets were combined to generate a consistent and comprehensive gene set in “maker” (v1.0) (Cantarel et al., 2008; Thrasher et al., 2014).

### Comparison of Genome

To compare the assembled leopard coral grouper genome to other already known Serranidae fish genomes, we used Lastz (v1.02) (Harris, 2007). These results were then plotted in the “circus” (v0.69) software (Krzywinski et al., 2009).

### Usage Notes

All contig sequences were assembled into chromosomes by using interaction information from the Hi-C sequencing data. Hence, we used 500 bp to represent the unknown gap sizes among contigs in the obtained chromosome sequences.

### Code Availability

The execution of this work involved using many advanced software tools. The settings and parameters for these are provided below.

Genome assembly: (1) *minimap2*+*miniasm*: all parameters were set to their defaults; (2) *racon*: all parameters were set to their defaults; (3) *pilon*: all parameters were set to their defaults; (4) *3d-dna*: -m haploid -s 4 -c 24 -j 10.

Genome annotation: (1) *ProteinMask*: -engine ncbi -noLowSimple -pvalue 0.0001; (2) *RepeatMasker*: -nolow -no_is -norna -engine ncbi -parallel 1; (3) *LTR_FINDER*: -w 2; (4) *RepeatModeler*: -database genome -engine ncbi -pa 9; (5) *TRF*: matching weight = 2, mismatching penalty = 7, INDEL penalty = 7, match probability = 80, INDEL probability = 10, minimum alignment score to report = 50, maximum period size to report = 2,000, –d –h; (6) *Augustus*: –uniqueGeneId = true –noInFrameStop = true –gff3 = on –strand = both –species = zebrafish; (7) *SNAP*: all parameters were set to the defaults; (8) *BLAST*: -p tblastn -e 1e-05 -F T -m 8 -d; (9) *tophat*: –max-intron-length 20000 -m 1 –solexa-quals -r 20 –no-coverage-search –mate-std-dev 20 –microexon-search -p 8; (10) *cufflinks*: -I 20000 -p 4; (11) *maker*: all parameters were set to the defaults.

Genome alignment: (1) *Lastz*: T = 2, C = 2, H = 2,000, Y = 3,400, L = 6,000, K = 2,200, –format = axt; (2) *Mcscan*: -a -e 1e-5 -u 1 -s 5.

## Results and Discussion

### Library Construction and Sequencing

After removing any redundant and low-quality reads, a total of 38.56 Gb (43.6X) clean reads were left, which included 18.14 and 20.42 Gb of reads from the 500- and 800-bp reads length via Illumina sequencing, respectively. After the k-mer analysis, all the clean reads were estimated to be 945 Mbp using the Genome Characteristics Estimation (GCE) software. A nanopore library was constructed and sequenced using the GridION X5/PromethION sequencer, which yielded 76.93 Gb of final contigs. The high-throughput chromosome conformation capture (Hi-C) library was sequenced by the Illumina HiSeq X10 platform (with 150 bp PE model). This Hi-C sequencing was done for chromosome-level scaffold constructions, yielding a total of 52.75 Gb of paired-end Hi-C reads generated, whose average sequencing coverage was 59.9X (Table 1).

**Table 1:** Sequencing data used for the leopard coral grouper's genome assembly.

**Sequencing**	**Insert**	**Total**	**Reads**	**Sequence**
**strategy**	**size**	**data (bp)**	**length (bp)**	**coverage (X)**
Illumina	500 bp	19,478,316,000	150	20.5
	800 bp	21,927,815,500	125	23.1
Nanopore	–	82,601,731,312	30,352	86.9
Hi-C	400 bp	56,639,825,400	150	59.9
Total	–	180,647,688,212	–	190.5

*The sequence coverage values were calculated based on the genome size estimated by the k-mer-based method*.

### Genome Assembly and Chromosome Anchoring

**Figure 2:**
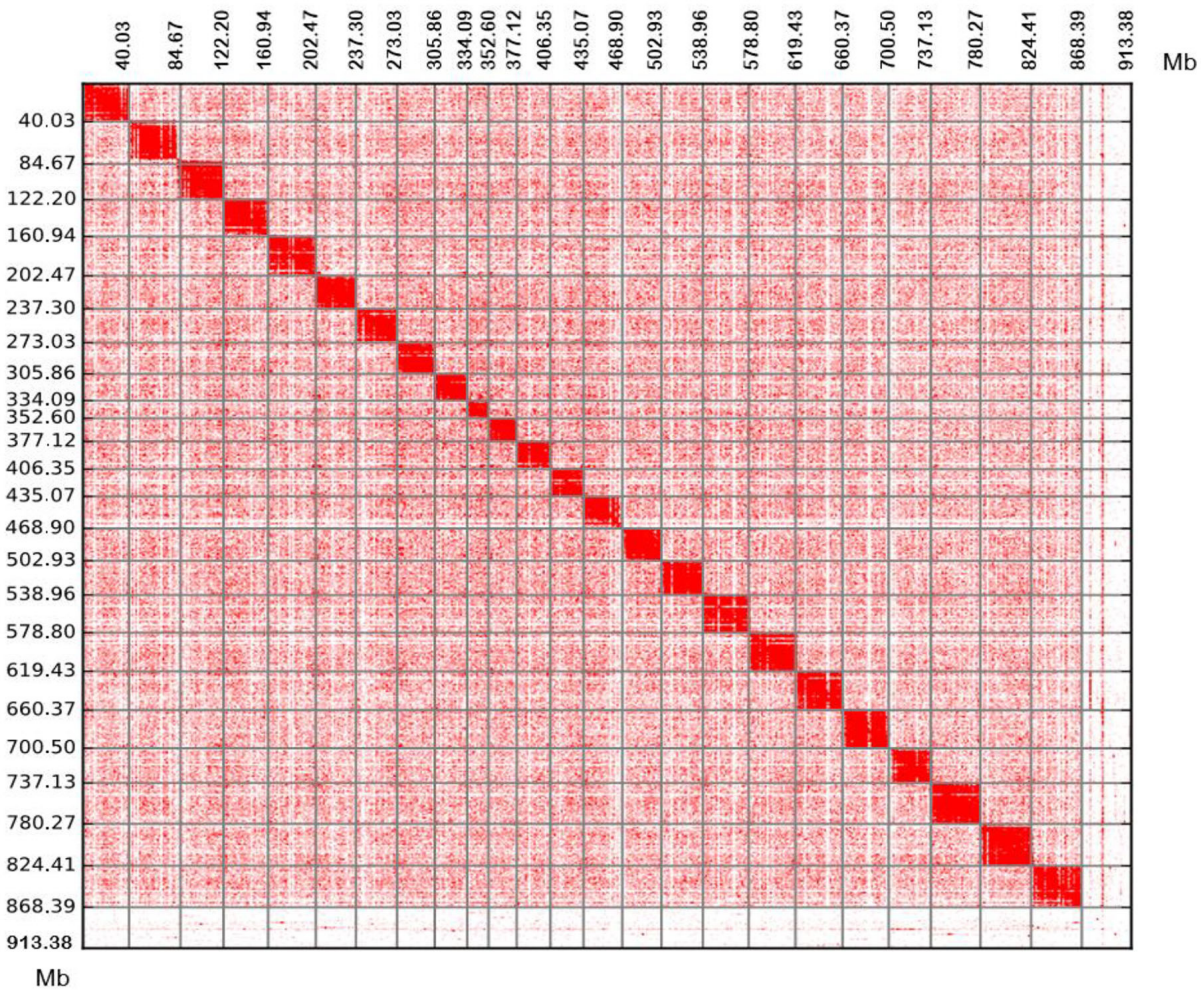
Chromosomal contact maps. The blocks represent the contacts detected between one location and another. The colour reflects the intensity of each contact, with deeper colouring used to indicate a higher intensity. Each number in the x-axis and y-axis means the genomic length (Mb).

We obtained an assembled genome of leopard coral grouper containing 1,526 contigs, whose total length was 912.66 Mb. The assembly covered 96.5% of the estimated genome regions. The contig N50 length was 1.42 Mb (Table 2). Through Hi-C data, 1,346 contigs were found anchored and orientated on 24 chromosomes, including 95.2% of genomic sequences; the results were consistent with previous karyotype analyses of the leopard coral grouper (Gao et al., 2015). The respective lengths of the 24 chromosomes ranged from 18.6 to 43.74 Mb (Tables 2, 3; Figure 2). Compared with other Perciformes fish, the genome assembly of *P. leopardus* shows a higher level (Table 4).

**Table 2:** Genome assembly statistics for the leopard coral grouper.

**Type**	**Scaffold**	**Contig**
Total length (bp)	913,382,752	912,657,252
Contig N50 length (bp)	40,038,452	1,412,334
Contig N90 length (bp)	28,399,218	217,765
Maximum length (bp)	43,747,248	7,817,432
GC content	39.4%	39.4%

**Table 3:** Summary of the assembled chromosomes of the leopard coral grouper.

**Chromosomes**	**Length (bp)**	**Number of contigs**	**Gene number**
Chr1	43,747,248	50	1,182
Chr2	43,338,506	56	987
Chr3	43,327,923	107	1,193
Chr4	41,481,500	58	1,182
Chr5	41,352,528	76	1,163
Chr6	41,116,667	69	1,043
Chr7	41,088,328	76	1,073
Chr8	41,017,068	80	1,033
Chr9	40,373,582	49	1,123
Chr10	40,147,833	80	1,187
Chr11	40,038,452	89	1,191
Chr12	38,795,244	67	1,148
Chr13	35,959,459	57	979
Chr14	35,875,708	36	902
Chr15	35,801,114	55	998
Chr16	34,844,746	42	877
Chr17	34,135,762	39	969
Chr18	33,903,067	68	980
Chr19	32,808,368	38	1,017
Chr20	29,357,699	40	782
Chr21	28,567,991	47	690
Chr22	28,399,218	40	644
Chr23	24,312,335	35	657
Chr24	18,601,276	30	453
Linked total	868,391,622	1,346	23,453
Unlinked total	44,991,130	180	1,246
Linked percent	95.2%	88.2%	94.9%

**Table 4:** The genome assembly statistics of several Perciformes fish.

**Species**	**Assembled genome size**	**Scaffold N50**	**Contig N50**	**Gene number**	**Mapping rate of chromosome**	**References**
*Dicentrarchus labrax*	675 Mb	5.1 Mb	53 kb	26,719	86%	Tine et al., 2014
*Epinephelus akaara*	1.135 Gb	46.03 Mb*	5.25 Mb	23,808	95.55%	Zhou et al., 2019
*Epinephelus lanceolatus*	1.086 Gb	46.2 Mb*	119.9 kb	24,718	98.4%	Ge et al., 2019
*Trachinotus ovatus*	647.5 Mb	5.05 Mb	1.80 Mb	21,915	99.4%	Zhang et al., 2019
*Larimichthys crocea*	669.78 Mb	6.55 Mb	282.69 kb	26,100	90%	Mu et al., 2018
*Lates calcarifer*	668.5 Mb	25.8 Mb*	1.06 Mb	22,184	87.8%	Vij et al., 2016
*Plectropomus leopardus*	913 Mb	40.04 Mb*	1.42 Mb	24,700	95.2%	This study

*The asterisk represents chromosome-level scaffold N50*.

Using the vertebrata_odb9 database, we found that 92.5% BUSCO genes were completely within the leopard coral grouper genome. We then aligned Illumina short reads to the genome using “bwa” (v0.7.15), finding that more than 94.25% of the reads were aligned to the reference genome, which demonstrated a high mapping ratio for the short-read sequencing data.

### Repeat Sequences and Gene Annotation

A total of 315.75 Mb (34.59% of the assembled genome) repeat sequences were thus identified. Among these repeat elements, DNA transposons were more abundant than any other types, accounting for 17.59% (160.56 Mb) (Table 5).

**Table 5:** Summary statistics for the annotated repeat sequences.

**Type**	**Repbase TEs**		**TE proteins**		***De novo***		**Combined TEs**	
	**Length (bp)**	**% in genome**	**Length (bp)**	**% in genome**	**Length (bp)**	**% in genome**	**Length (bp)**	**% in genome**
DNA	34,078,348	3.73	1,715,926	0.19	147,891,061	16.20	160,562,959	17.59
LINE	17,746,417	1.91	13,612,807	1.49	60,770,280	6.65	82,561,848	9.04
SINE	2,149,356	0.23	0	0.00	1,640,938	0.18	2,841,026	0.31
LTR	10,757,322	1.17	4,273,499	0.47	61,117,147	6.69	71,106,208	7.79
Other	27,455	0.003	72	0.00	0	0.00	27,527	0.003
Unknown	0	0.00	0	0.00	63,867,205	6.99	63,867,025	6.99
Total	58,320,329	6.39	19,596,947	2.14	285,544,317	31.28	315,750,864	34.59

A total of 24,700 protein-coding genes were predicted. The average number of exons per gene and average gene length were 9.7 and 1,777 bp, respectively (Table 6). In all, we were able to annotate 24,014 genes in at least one of the databases; hence, in this way, 97.22% of leopard coral grouper genes were functionally annotated (Table 7).

**Table 6:** Summary statistics of predicted protein-coding genes.

**Gene set**		**Number**	**Average transcript**	**Average CDS**	**Average exons**	**Average exon**	**Average intron**
			**length (bp)**	**length (bp)**	**per gene**	**length (bp)**	**length (bp)**
*De novo*	Augustus	27,740	15,893	1,417	8.3	170	1,980
	SNAP	72,047	19,338	967	6.85	141	3,137
Homolog	*Takifugu rubripes*	19,388	16,833	1,638	10	163	1,682
	*Lepisosteus oculatus*	15,494	21,220	1,714	10.8	157	1,973
	*Gasterosteus aculeatus*	28,411	14,597	1,475	9.3	157	1,572
	*Oreochromis niloticus*	26,405	19,590	1,786	10.7	165	1,817
	*Oryzias latipes*	20,987	15,042	1,474	9.1	160	1,656
	*Danio rerio*	17,176	22,791	1,576	9.6	163	2,448
	*Epinephelus tauvina*	23,026	17,077	1,636	9.7	168	1,766
	*Lates calcarifer*	24,253	16,883	1,776	10	182	1,732
RNA-seq	Cufflinks	19,890	6,176	1,398	5.1	274	1,166
Maker		24,700	16,883	1,777	9.7	182	1,732

**Table 7:** Statistics for the functional annotation of protein-coding genes.

**Database**	**Number**	**Percent (%)**
NR	23,986	97.10
SwissProt	19,994	80.95
Interpro	21,518	87.11
KEGG	21,277	86.14
At least one database	24,014	97.22
Total	24,700	100

*Note that “at least one database” refers to those genes with at least one hit among the multiple databases searched*.

### Comparison With Other Serranidae Fish Genomes

**Figure 3:**
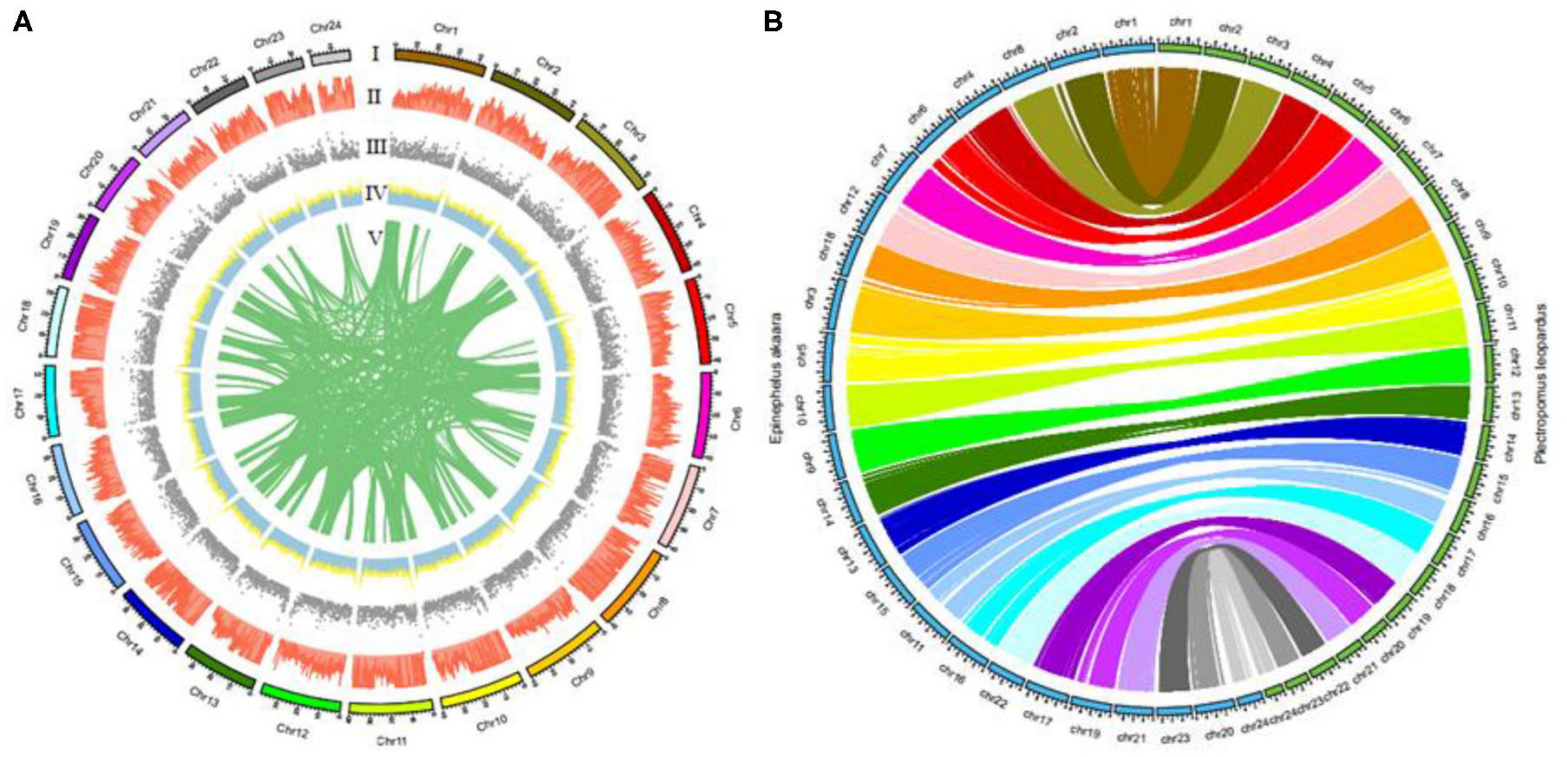
Circos atlas representation of chromosome information. **(A)** (I) Length of each chromosome. (II) Density of SNP distribution in each 100-kb genomic interval. (III) Density of gene distribution in each 100-kb genomic interval. (IV) GC content of 100-kb genomic intervals. (V) Schematic presentation of major interchromosomal relationships in the leopard coral grouper genome. **(B)** Circos diagram representing syntenic relationships found between the leopard coral grouper and the red-spotted grouper.

We recently used Lastz (v1.02) to successfully compare the leopard coral grouper genome to the red-spotted grouper *(Epinephelus akaara*) genome (Ge et al., 2019). Figure 3A summarizes the distribution of SNPs, genes, GC content on 100-kb genomic intervals, as well as the interchromosomal relationships of our assembled leopard coral grouper chromosomes. The genomic sequences of the red-spotted grouper showed evidence of synteny to the leopard coral grouper's genome. We found that the 24 chromosomes of the red-spotted grouper had a clear one-to-one relationship to the leopard coral grouper's chromosomes (Figure 3B). According to these results, we therefore anticipate that the leopard coral grouper genome will contribute to the study of genome evolution in the Serranidae family members.

## Data Availability Statement

The genome assembly sequences and predicted gene were deposited in at CNGB under the accession CNA0007316. The Illumina genomic sequencing reads, Nanopore long reads, Hi-C data, and RNA-seq reads were deposited in CNGB under the accession CNP0000859.

## Ethics Statement

The animal study was reviewed and approved by Institutional Review Board on Bioethics and Biosafety of BGI (No. FT 18134).

## Author Contributions

FC, JLu, and XY contributed to the study design. YW, SF, JLi, WT, ML, LL, and HH contributed to the fish culture and sample preparation. XZ, YW, and XW performed the bioinformatics analysis. JLu, XY, XZ, and XW wrote the paper. All authors read and approved the final manuscript.

## Conflict of Interest

The authors declare that the research was conducted in the absence of any commercial or financial relationships that could be construed as a potential conflict of interest.
